# The Awakening of the People

**Published:** 1918-08-10

**Authors:** 


### THE GREAT WAR.

### X.—THE AWAKENING OF THE PEOPLE.

THE fourth anniversary of Britain's declaration of war against Germany was celebrated on the 4th inst. throughout Great Britain, the Empire and the United States of America. The American press, and the speeches of eminent men in the United States, testify that the American people to-day, as the *New York Tribune* puts it, "not only admire all that Britain and the Empire have done, but also understand it." The words of affection and gratitude cabled from representative men—politicians, representatives of science, art, and culture, and of the United States press—have brought us nearer, if that be possible, to our cousins across the Atlantic. They were most welcome and deeply touching too. We were in America at the Presidential Election in the fall of 1916, when it was by no means clear what view the citizens of the United States took of the war, and where their sympathies lay. Indeed, evidence was forthcoming which pointed to the active German influence at work almost everywhere, and President Wilson, before the result of the Presidential Election was known, was held to be an ardent German sympathiser whom the Kaiser had instructed the German population of the United States to support with all their forces. In upholding the cause which fights for freedom, in President Wilson's words, she prays Almighty God to purify the hearts of all our peoples to see and love the truth, to accept and defend all things that are just and right . . . beseeching Him that He will give victory to our armies as they fight for freedom, wisdom to those who take counsel on our behalf . . . and steadfastness to make sacrifice to the utmost in support of what is just and true." It has been our privilege to be brought into contact with some of the soldiers from America and their officers of various ranks, to see their work and to learn their views. These experiences have been most encouraging and helpful, for they give one the true inwardness of American feeling as testified by the press and the speakers in the United States on the 4th inst., and demonstrate that all that is best in the Anglo-Saxon nations to-day is united heart and soul in upholding the cause upon the success of which the world's well-being depends. Every well-wisher of the united nations may gratefully thank God, and take courage that this union has come to pass with a completeness and suddenness which point to the hand of Almighty God, and as the Archbishop of Canterbury declared, "this unity seals the conviction that the trust the Anglo-Saxon nations have undertaken together they solemnly believe is divinely given.

Remembrance Day throughout Great Britain was celebrated by special services of thanksgiving and intercession and by public meetings throughout the country. Their Majesties the King and Queen, with both Houses of Parliament, went to say their prayers at St. Margaret's Westminster. We wrote of St. Margaret's in the *Westminster Gazette* of the 22nd ult., and all we ventured to state was exemplified in the service on the 4th inst., which, as is usual at St. Margaret's, was as simple as that in any large parish church of the best. One marked difference was the reading of the lessons by the Speaker and the Lord Chancellor, and the presence of the Archbishop of Canterbury as the preacher. His text, Exodus xxii. 7, "Thou shalt not take the name of the Lord Thy God in vain" was well chosen. The lessons of the hour which he drew from it for all nations and people were forcibly put, and we cannot do a greater kindness to everyone interested in the war, its meaning and successful completion, than to suggest to them that they should obtain a copy of his sermon and preserve it for periodical study and reference. It was indeed a tremendous decision when the representatives of Britain decided on August 4, 1914, to declare war against Germany, a decision for which we were, and are, answerable to God and man.

The service at St. Margaret's Church last Sunday was without precedent, for it was the first occasion in the history of our nation that the King and Queen and the two Houses of Parliament have joined officially in one solemn act of prayer, confession, thanksgiving, commemoration, and resolve. In the reverently acknowledged presence of the living God in our worship and meetings on the 4th inst. we recalled and asserted afresh the place we take, the line we follow, in the mightiest war ever waged on earth. Take this gathering of the people at St. Margaret's, at the cathedrals and churches of all denominations throughout the country, at the meetings in Hyde Park or in every large place where people usually gather throughout the metropolis, and throughout the cities, towns, and villages of Great Britain, the Empire, and in many parts of the United States where services were held, and the proceedings demonstrate that those present were Christians, members of Christ's society, and proved that the place of the Allied nations in the war is deliberate. Those we attended, and especially the great congregation assembled in Hyde Park at the service conducted by the Bishop of London, and the after service, the dedication of the shrine to our dead heroes, with its splendid demonstration of flowers, and the many and continuous incidents including the bringing of sometimes one flower, sometimes more, by boys and girls in memory of their fathers and brothers, went straight to the heart. It may be truly said of the services on Sunday last, both in places of worship and in the open air, that they were most impressive and reverent, and that the whole conduct of the people throughout that day, wherever they might be assembled, testified to their hearts

☛ Previous articles appeared on Feb. 9, 23, March 9, 23, April 6, 20, May 4, 25, June 8 and 22 pp. 247, 248, and 250.

**The Great War**—(*continued*).

being in the celebration of the day and all it meant to the people of the Empire and to the people of the nations who, with Great Britain, constitute the Allies. There was indeed and truly an atmosphere of recognition by all of the presence of the living God, and it is clear that men and women through this war have been awakened to a sense of their spiritual nature, their dependence on Almighty God, the efficacy, power, and comfort of prayer, and the presence in their lives of new forces, a new hope, and a great resolve, which, under God's guidance, will, we are confident, form a new world after this war. Then every man, woman, and child will have greater opportunities and widespread security, and to every one of them who resolves to attune their lives to the highest standard they can conceive, the future is full of promise, and in God's mercy will bring the splendid realisation of a new life.

The evidences which surrounded ministers of all denominations who have eyes to see, a heart to feel, and intelligence to understand the happenings on Remembrance Day 1918 cannot, surely, have left the shepherds of souls unmoved and indifferent to the widespread and continuously extending recognition of the Living God by all classes in our midst? The people generally will look to them for evidence of their own awakening and quickened sense of their spiritual natures to the uplifting and helping of their congregations. Such evidence will be strengthened, then, by their continuous efforts to help to give to the atmosphere of every place of worship throughout the United Kingdom a spiritual vitality which will cause every reverent worshipper who enters it to feel and recognise that it is the House of the Living God.

### Britons within the Metropolis.

The quietness of the people everywhere, their settled demeanour and assurance of gait and appearance throughout London, were remarkable. They seemed to have caught some of the spirit and much of the manner of our fighting men, and it may be said with truth that the types of fighting men to be met with throughout London on that day were in tune with the people, and made it a joyous and restful experience to be amongst them, to study them, and to enter fully into the spirit of assured confidence and awakening to the living God which undoubtedly possessed the majority. The gatherings in the parks and open places, where the same characteristics were exhibited by those present, the majority of whom had come to say their prayers at the services widely held in the open air on that day, set the note of the occasion. There was a certain air of solemnity which undoubtedly exhibited how deeply conscious the overwhelming majority of the people were of the significance of Remembrance Day and its celebration. The speakers, too, were in tune with the people, and much fine and moving eloquence resulted. The congregations were gatherings of members of every religious sect, who with the speakers represented all types of churches comprised by the Christian faith. In Hyde Park, in addition to the Lord Bishop of London, the Rev. J. C. Spurr, the minister of Regent's Park Chapel, a notable centre of religious thought, who had been interned in Switzerland, and had had some trying experiences, spoke with great power and force. He, like the Bishop and other speakers, suffered from the absence of reporters, to the great loss of the citizens, and we hope that Mr. Spurr may be moved to publish his speech, as its general circulation could not fail to do good work in many ways at the present time. He paid a glowing tribute to *Punch*, and we heartily share his encomium of the indebtedness of all classes of people throughout the English-speaking nations to Sir Owen Seaman, Mr. Bernard Partridge, Mr. Leonard Raven-Hill, and other members of the *Punch* staff for the international work they have done in bringing home to every single person the true inwardness of this war, and the real aims of the men responsible for it. Invaluable, too, were *Punch's* exposure of the foibles and follies of men at present uninterned and resident in Great Britain, who claim to be Britons, but whose conduct demonstrates them to be suffering from senile decay or treasonable degeneracy. They are the possessors apparently of one idea, which is to do all in their power to help the Germans, and to render it impossible for the Allies, if they can so provide, to obtain the only peace which will uphold and enforce those principles, the maintenance of which alone can guarantee and secure the well-being of the world. The overwhelming majority of Britons fail to understand why these senile and reprehensible degenerates are not interned, or at least silenced, by having it taken out of their power to further organise mischievous movements and to circulate so-called literature, the only effects of which must be to injure the cause of the Allies, and to leave open the door to the perpetuation of the militarist menace and the keeping alive of its organisation in Germany as a centre and assured re-creator, if they have their will, of all the evils, in an aggravated form, from which the world at present suffers.

### Our American Brothers.

The articles on the Great War, of which the present is one, which we are publishing, aim at being a faithful, outspoken record of facts and happenings which should possess a permanent interest for all time. It is a pleasure, indeed, to turn from the miserable creatures who spend their lives in sowing sedition and working against the welfare of the nations and their peoples to the wholesome, invigorating, and manly atmosphere which surrounds the men from the United States who are coming over every day in thousands to fight for the cause of right and the freedom of the world. The 20,000 people or more who attended the service in Hyde Park on the 4th inst. found Dr. Sherwood Eddy a splendid specimen, both in oratory and in person, of our American brothers

**(Continued on page 409.)**

### THE GREAT WAR— (*Continued from page* 413.)

at their best. He gave a most stirring address, delivered with a clearness and force which held and moved his hearers and won their sympathies. It was, in fact, a great effort, full of information of the deepest interest to Britons as well as Americans, and we have great pleasure in publishing the substance of it, on page 414. Dr. Sherwood Eddy has been the Secretary for Asia of the International Committee of Young Men's Christian Associations, whose head office is at 347 Madison Avenue, New York. He has been asked to go to the Front to help in the work there and in Britain. We welcome his presence and co-operation, and commend him with confidence to the hospitality and friendship of every resident in Great Britain, and every assembly where Britons are to be found.

